# Evaluation of the Simple One-Step (SOS) stool method for Truenat MTB plus and Xpert MTB/XDR assay

**DOI:** 10.1128/jcm.01040-25

**Published:** 2025-12-10

**Authors:** Petra de Haas, Joel Kabugo, Joanita Namutebi, Edine Tiemersma, Hasfah Ssentamu Nakato, Charles Manyonge, Daniel Kisakye, Tayebwa Atwine Mukama, Dorothy Nassozi, Andwele Mwansasu, Moses Joloba, Eveline Klinkenberg

**Affiliations:** 1 KNCV Tuberculosis Foundation 26102, The Hague, the Netherlands; 2 Supra National Refence Laboratory, Kampala, Uganda; 3 IDDS Project, ICF, Washington, DC, USA; 4 Connect TB, the Hague, the Netherlands; Consultancy services provided for USAID TB team, Washington, DC, USA; The University of North Carolina at Chapel Hill School of Medicine, Chapel Hill, North Carolina, USA

**Keywords:** stool, Truenat, Xpert-Ultra, children, TB diagnosis

## Abstract

Stool is a recommended sample for the diagnosis of *Mycobacterium tuberculosis* (MTB) using Xpert MTB/RIF (Ultra) (GXU) in children, and the Simple One-Step (SOS) stool method is one of the recommended processing methods. We investigated modifications to the protocol of the SOS stool method to make it fit for testing stool with Truenat MTB Plus and MTB-RIF Dx (Truenat) and Xpert MTB/XDR (GXX). Experiments were conducted using stool spiked with different MTB concentrations and using modified versions of the SOS stool processing method. The established protocol was then validated on routine clinical stool samples from presumptive TB patients, comparing the performance of Truenat and GXX against GXU. The concordance for MTB detection between GXU and Truenat using 50, 100, and 150 mg of stool was 100%. Truenat did not provide valid results at 300 mg. In routine stools, concordance for MTB detection was 89.3% and 90.6% when comparing Truenat-100 mg to GXU-100 and 600 mg, respectively. For rifampicin detection, 44.8% of results were indeterminate on Truenat, but none on GXU (except Trace calls). GXX stool testing gave valid results, although for low bacterial loads, GXX less often detected MTB. We conclude that stool can be tested on Truenat to detect MTB using the SOS stool Xpert method adapted to the test composition of Truenat. In addition, the SOS protocol, as originally developed for GXU, can be used for GXX. This provides opportunities to expand access to rapid molecular testing to detect TB and resistance to first- and second-line anti-TB drugs for children and adults who cannot produce sputum.

**IMPORTANCE:**

Following World Health Organization recommendations, countries commenced stool testing on GeneXpert as the primary test to improve tuberculosis (TB) case finding among children who cannot produce sputum. To further expand access to rapid molecular testing, we adapted the Simple One-Step (SOS) stool method for GeneXpert to the test kit composition of the Truenat platform through a series of laboratory experiments. Truenat is being introduced on a large scale by countries for near point-of-care testing as it can operate better in more remote settings. As part of our experiments, we also concluded that stool can be tested with the Xpert MTB/XDR cartridge using the original SOS protocol. Our findings further expand access to rapid molecular testing to detect TB and resistance to diverse anti-TB drugs for children and adults who cannot produce sputum. Increased access to TB testing in these vulnerable populations will support TB case finding efforts and timely and appropriate treatment.

## INTRODUCTION

The diagnosis of pulmonary tuberculosis (TB) in children is challenging because they often present with nonspecific signs and symptoms, and laboratory confirmation of their disease is difficult due to its paucibacillary nature and the difficulty in obtaining sputum (1, 2). Since 2020, the World Health Organization (WHO) has recommended the use of Xpert MTB/RIF (GX) and Xpert MTB/RIF Ultra (GXU) on stool as an initial diagnostic test for the detection of TB and resistance to rifampicin (RIF) in children with signs and symptoms of pulmonary TB (2, 3). The Simple One-Step (SOS) stool processing method is proposed as the simplest and lowest-cost option (4, 5). The SOS stool method requires the same supplies and equipment as used for GX and GXU testing of sputum, while demonstrating similar sensitivity and specificity compared to a more complex method (2, 4–6). Since 2020, besides GXU, another rapid molecular WHO-approved diagnostic (mWRD) for the detection of TB and RIF resistance, the Truenat MTB Plus, combined with MTB-RIF Dx assay (Molbio Diagnostics, India) (Truenat), has been recommended for screening (3). This assay, like GXU, is recommended for placement at the peripheral (or community) level as an initial diagnostic and can diagnose TB and RIF resistance. Testing for TB and RIF resistance on the Truenat platform has shown comparable results to the GeneXpert platform in routine clinical settings, including among people living with HIV (7, 8). The benefit of the Truenat above the GeneXpert platform is that this technique can be implemented more easily in primary healthcare facilities, as it requires minimal infrastructure (3). Truenat is now being introduced on a larger scale by countries for near point-of-care testing, especially in more remote places where GX and GXU testing is challenging due to infrastructural and power limitations (9, 10).

Besides bacteriological confirmation of *Mycobacterium tuberculosis* (MTB) and confirmation of RIF resistance, it is also important to test for resistance to other anti-TB drugs to ensure appropriate treatment initiation. WHO recommends the use of the Xpert MTB/XDR (GXX) assay on sputum for the initial detection of resistance to isoniazid, fluoroquinolones, ethionamide, and amikacin in people with bacteriologically confirmed pulmonary TB rather than culture-based phenotypic drug susceptibility testing (3). The GXX assay is not yet evaluated for testing using stool, though anecdotal evidence exists on its use with stool and the SOS processing method (11, 12). Since GXX and the Truenat platform are now being introduced on a large scale by countries for near point-of-care testing, it would be opportune to evaluate the use of stool on both the Truenat platform and the GXX assay.

This manuscript describes the results of a series of laboratory experiments performed on stool spiked with *Mycobacterium tuberculosis* bacteria to establish the SOS protocol for stool-based testing adapted to the Truenat platform and testing of stool using the GXX assay. Furthermore, it describes the results of using the established protocol on routine stool samples from persons with presumptive and confirmed TB.

## MATERIALS AND METHODS

### Study design and setting

This study was conducted in two phases: phase 1 was a laboratory study consisting of a series of experiments using stool spiked with MTB, and phase 2 was a cross-sectional study in which routine stool samples were tested using the (modified) SOS protocol established in phase 1. Phase 1 aimed to define the stool processing protocol for the Truenat platform and the XDR assay, while phase 2 aimed to compare the performance of stool Truenat and GXX testing against SOS stool-based testing on GXU. The study was conducted at the National Tuberculosis Reference Laboratory (NTRL) in Kampala, Uganda, which also serves as a WHO Supranational Reference Laboratory.

### Phase 1—experiments to establish the protocol for stool on Truenat and GXX

**Fig 1:**
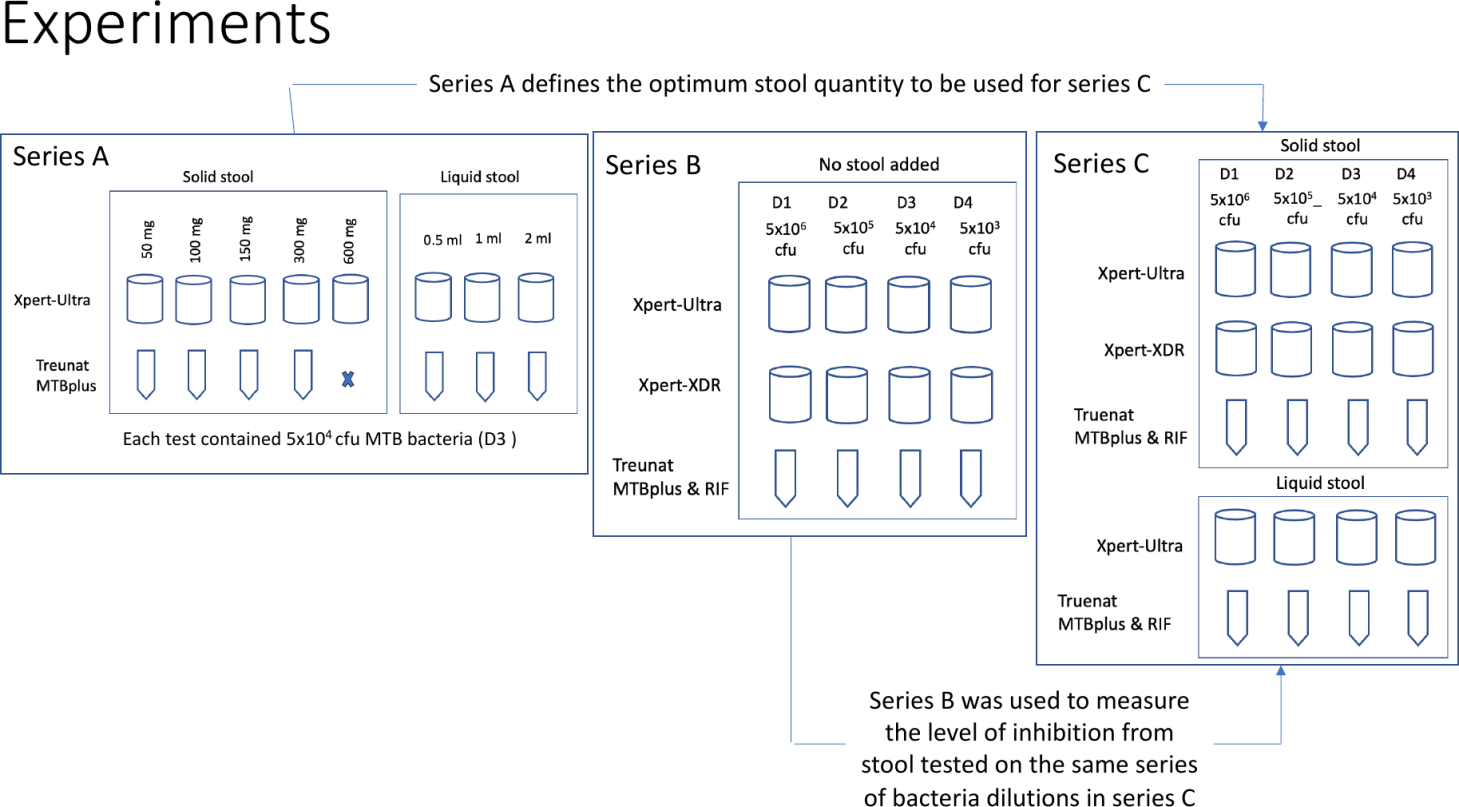
Schematic overview of the three series of experiments in phase 1 using stool spiked or not spiked with different concentrations of MTB bacterial suspensions. cfu, colony-forming units.

This phase consisted of three series of laboratory experiments, series A, B, and C (Fig. 1). The experiments of series A were designed to determine the optimum amount of stool to be tested. For solid stool, quantities of 50, 100, 150, 300, and 600 mg were tested, and for liquid stool, 0.5, 1, and 2 mL were used. Although the initial WHO-recommended amount for the SOS stool method is 800 mg (4, 5), recent work has shown that SOS stool works best (e.g., less non-determinant results) with 300–600 mg of stool (13); therefore, we decided to test 600 mg as the reference. The test bottles used in these experiments were all spiked with the same concentration of MTB suspension. Test series A experiments were conducted using GXU and Truenat MTB Plus. These were not performed on GXX due to the limited test capacity of the 10-color module and the availability of cartridges. In addition, we assumed that the within-cartridge technology is the same for GXX as for GXU, which means these tests behave similarly when using stool processed with the SOS stool method. In series B, a series of different concentrations of MTB suspensions was tested using GXU, GXX, and Truenat without adding stool to create a reference for series C. Then, in series C, the same series of MTB suspensions was tested using the same assays but adding the optimum amount of stool defined in series A to provide insights into the level of inhibition of the PCR technology when adding stool to Truenat and GXU at different concentrations.

For each combination of test variables, the assay, amount of stool, and concentration of MTB bacteria added (called “test condition” hereafter), five runs were conducted. If one of the runs resulted in “invalid,” “error,” or “no result,” an additional run was performed until valid test results for five runs were available. If three consecutive runs resulted in non-deterministicte results, no further runs were conducted for that condition to save resources. There were a few exceptions: for GXX, series C consisted of four runs only due to the limited capacity of the 10-color module and the availability of cartridges. In addition, for liquid stool, fewer runs were conducted than planned due to the overall high non-determinate rate observed. Consistency between the five valid runs from the same test condition was measured using the mean and standard deviation of the cycle threshold (Ct) value per probe. Additional test runs were performed if, for a given test condition, the standard deviation of the Ct value around the mean of the five runs of any probe varied by more than three cycles.

Based on the results of the three series of experiments, the SOS stool processing protocols for Truenat and GXX were established for further evaluation in phase 2.

#### Preparation of stool mixture

To conduct the experiments, leftover stool samples collected from children (<10 years old) who tested MTB negative by GXU at the nearby health facility were used. The stool was retested at the NTRL to confirm the initial MTB-negative GXU result. It was estimated that approximately 200 g of solid stool and 100 mL of liquid stool would be needed to prepare the required aliquots of stool for all the experiments planned. Three stool specimens were mixed and homogenized to obtain enough stool for all tests. To obtain liquid stool, 20 g of the solid stool mixture was homogenized in 80 mL of 0.8% sodium chloride (saline) by carefully stirring using a wooden stick.

Both prepared stool mixtures (solid and liquid) were stored at 2°C–8°C in between experiments. All experiments were conducted within 10 days to avoid the stool mixtures from deteriorating in quality, which might affect the study results (13).

#### Preparation of MTB serial dilution suspensions

Four MTB serial dilution suspensions were prepared using the well-characterized H37Rv (ATCC 27294) strain that had been grown on the mycobacteria growth indicator tube (MGIT) media. Within the biosafety level 3 setting, the MGIT suspension was heat-inactivated (30 minutes at 80°C) and subsequently turbidity-adjusted through dilution with saline to approximately 0.5 MacFarland (i.e., 1.5 × 10^8^ cfu/mL) and labeled as D1 (14). Using this suspension, subsequent serial dilution suspensions D2 (2.5 × 10^7^ cfu/mL), D3 (2.5 × 10^6^ cfu/mL), and D4 (2.5 × 10^5^ cfu/mL) were prepared; see Table S1 in the supplemental material.

The MTB serial dilution suspensions were used to spike the 8 mL sample reagent (SR) bottle for GXU and GXX or the 2.5 mL lysis buffer (LB) bottle for the Truenat test. To allow comparison of the test results between the different assays, we ensured that after processing the stool, a similar number of bacteria was transferred into the cartridge. Therefore, the exact volume of MTB suspension to be added to the SR or LB bottle was calculated to obtain equal MTB concentrations per assay; see Table S1.

For experiment series A, the number of MTB bacteria that was transferred to the Truenat or GXU cartridge was 5 × 10^4^ cfu (using D3). For experiment series B and C, the number of MTB bacteria transferred to the Truenat, GXU, or GXX cartridge was 5 × 10^6^ cfu (using D1), 5 × 10^5^ cfu (using D2), 5 × 10^4^ cfu (using D3), and 5 × 10^3^ cfu (using D4); see Fig. 1 and Table S1.

#### Stool processing

**Fig 2:**
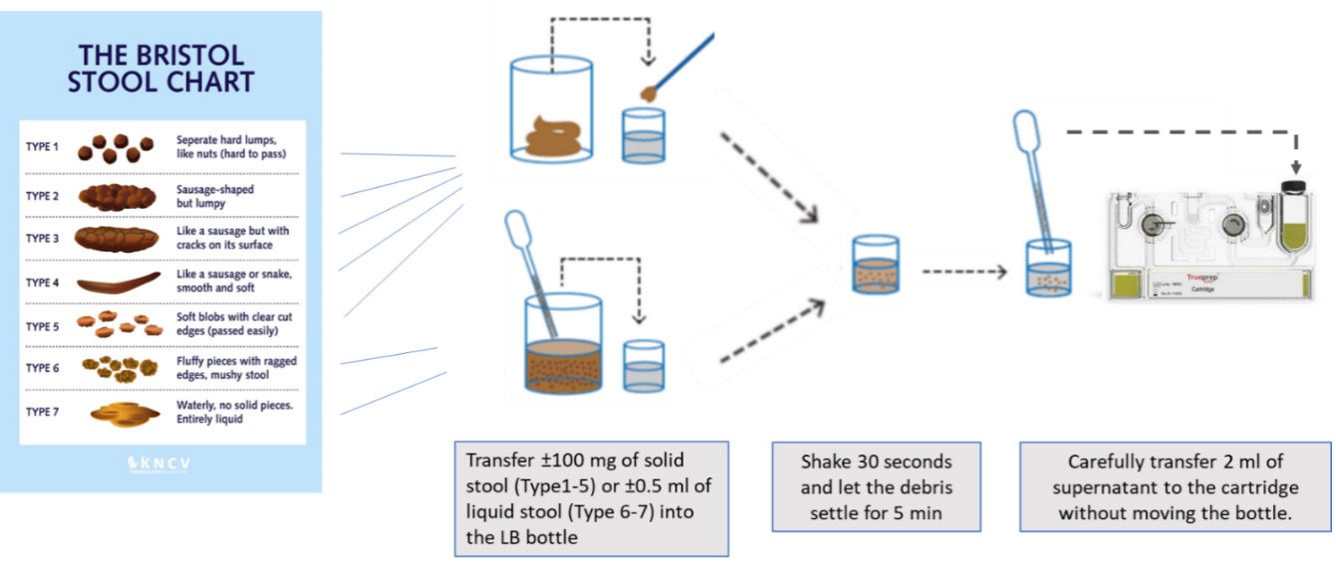
Schematic overview of the proposed Simple One-Step stool processing method for the Truenat assay.

For all experiments, the stool sample was processed using the SOS stool method as described by de Haas et al. (4), with adaptation envisioned to match the test composition of the Truenat assay, see Fig. 2. Briefly, for solid stool, the required quantity of stool (as per the experiments designed) was added to the 2.5 mL LB bottle using a wooden stick. For liquid stool samples, the volume of stool was directly transferred into the LB bottle using a balloon pipette, without removing any buffer. The stool/LB mixture was shaken by hand for 30 seconds and incubated at room temperature for 5 minutes. After incubation and sedimentation of the stool debris, 2 mL of the supernatant was transferred into the cartridge. After the cartridge run finished, the DNA eluate was removed, and 5 µL was transferred to the Truenat MTB Plus micro-PCR chip. If tested MTB positive, 5 µL of the same DNA eluate was transferred to the Truenat MTB-RIF Dx micro-PCR chip according to the instructions of the manufacturer (15).

### Phase 2—verifying established protocol for stool Truenat and GXX

Phase 2 involved a head-to-head comparison by testing quantities of stool as defined in phase 1. Aliquots taken from the same stool specimen from routine patients (children and adults) were processed using the standard SOS stool method for GXU on 600 mg (GXU-600 mg) and 100 mg of stool (GXU-100 mg) and GXX on 600 mg (GXX-600 mg) and 100 mg of stool (GXX-100 mg). In addition, an adapted SOS method established in phase 1 was used for Truenat on 100 mg of stool (Truenat-100 mg).

#### Collection of routine stools

For phase 2, between December 2023 and March 2024, we requested leftover stools from presumptive TB patients (both children and adults) tested in routine clinical settings through GXU at the site of collection. The aim was to include at least 30 GXU MTB-positive stools and a minimum of five stools that were tested GXU MTB negative. In practice, more stools were included, as not all routine test results were available immediately. To avoid missing inclusion of MTB-positive samples, all received stools were included in the study. The selected leftover stool samples were transported within 24 hours to the NTRL. Stool specimens had to be >300 mg to be eligible for inclusion. After arrival, aliquots from each stool sample were processed using the SOS stool method or an adapted version thereof, as defined in phase 1 and tested using GXU, GXX, and Truenat. If the initial test resulted in “invalid,” “error,” or “no result,” the test was repeated once from the same stool. First, the runs for GXU-100 mg and Truenat-100 mg were completed, followed by Truenat MTB-RIF Dx. Then, based on the available leftover sample, runs for GXU-600 mg were completed. Testing on GXX was performed only for those specimens with MTB detected on GXU and when sufficient stool was available. Stools with MTB detected on GXU-100 mg continued as GXX-100 mg, and those with MTB detected on GXU-600 mg continued as GXX-600 mg.

#### Data collection

Standardized data collection forms were created to capture the demographic data and test results per test condition. The data were manually captured on paper forms and entered into a REDCap (version 14.3.11) data collection tool designed specifically for this study. All test results were double entered, and discordances were checked against the collection forms for corrections. For phase 1, per test run, a unique barcode was assigned. Per test condition, the stool type (solid/liquid) and quantity of stool, dilution of MTB suspension (D1–D4), diagnostic assay used (Truenat/GXU/GXX), semi-quantitative test result, Ct values of all probes, initials of the person who performed the test, and date of testing were captured. For phase 2, a unique sequential number was assigned for each stool sample included in the study. No patient names were provided with the leftover stools; the only demographic variables collected were age, sex, and HIV status. We also collected information on the stool consistency, semiquantitative test result, and the corresponding Ct value of all probes.

#### Data analysis

All data were exported from the REDCap (Research Electronic Data Capture) tool (Vanderbilt University, USA) into MS Excel (Microsoft, USA) and Stata SE version 15.1 (Stata Corporation, College Station, TX, USA) for further data compilation and analysis. The average and standard deviation of the Ct values were calculated for each series of runs. The semi-quantitative GXU, GXX, and Truenat results, together with the actual Ct value of the target probes, were used to compare the sensitivity and potential inhibition of stool on the PCR technology across assays.

## RESULTS

### Phase 1—experiments to establish the protocol for stool on Truenat and GXX

A total of 179 test runs were conducted: 80 on GXU, 63 on Truenat, and 36 on GXX. To determine the optimum quantity of stool (series A), 62 runs (44 for solid stool and 18 for liquid stool) were conducted. To determine the effect of stool on the test (series B and series C), 60 runs were conducted without stool and 57 runs with stool (all solid stool). This is the total number of test runs conducted, including repeat runs, to reach a total of five valid runs per test condition. All test runs were completed within 10 days after preparing the stool mixture.

#### Determining the optimum quantity of stool for phase 2

For GXU, quantities of solid stool of 50, 100, 150, and 300 mg provided each five valid test runs, while 600 mg of solid stool initially provided four valid runs, with one run resulting in an error (code 2008). Therefore, a sixth run was completed to achieve five valid runs. For GXU on liquid stool, the 0.5 and 1 mL provided five valid test runs, whereas for 2 mL, three initial runs resulted in an error (code 2008), and further testing was stopped. For Truenat, the stool quantities of 50, 100, and 150 mg each yielded five valid test runs. For 300 mg of stool on Truenat, three runs were conducted, but all resulted in an error (code E03), and further testing was stopped. We observed that when adding 300 mg of stool, the LB became saturated, and it was not possible to remove a clean supernatant to transfer to the cartridge for testing; therefore, no runs were done to test 600 mg of stool for Truenat. For the liquid stool, none of the test conditions (0.5, 1, and 2 mL) resulted in valid test runs on Truenat, and no runs were conducted after two initial runs each for 0.5 and 1 mL, while for 2 mL, just one run was completed.

**Fig 3:**
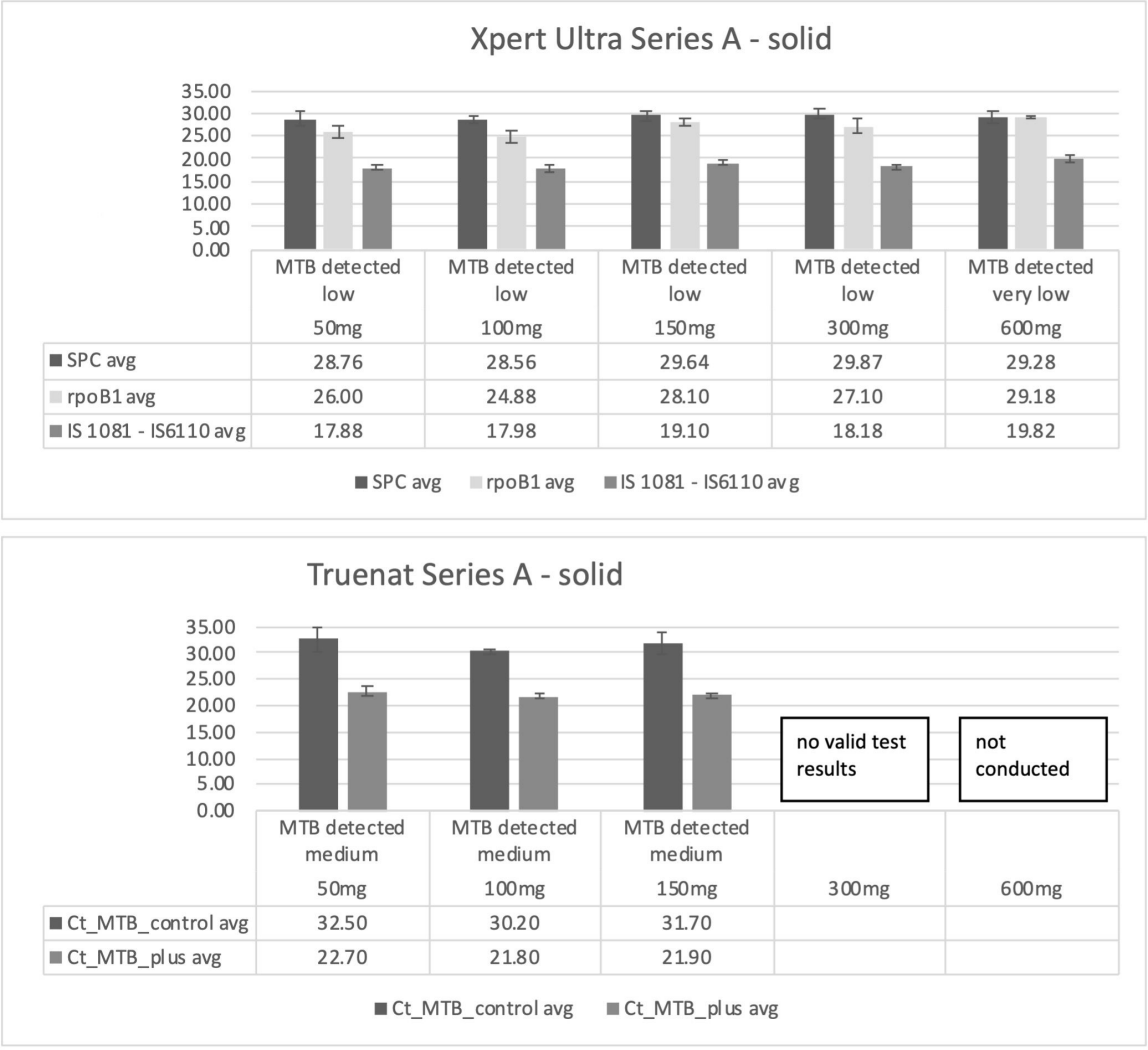
Average Ct values of five runs (with standard deviation) for the different target probes using various quantities of solid stool spiked with MTB (dilution D2) for both Xpert-Ultra (GXU) and Truenat MTB Plus. avg, average;.

Figure 3 presents Ct values, averaged over five valid runs per quantity of stool, of GXU-rpoB1, GXU-IS1081-IS6110, and Truenat-MTB Plus. The average Ct value ranged from 26.00 (50 mg) to 28.10 (150 mg) to 29.18 (600 mg) for GXU-rpoB1, from 17.88 (50 mg) to 19.10 (150 mg) to 19.82 (600 mg) for GXU-IS1081-IS6110, and from 22.70 (50 mg) to 21.90 (150 mg) for Truenat-MTB Plus. There was no significant change in Ct values when more stool was added. However, the average Ct values obtained with the Truenat-MTB Plus probe were slightly lower than those for GXU, resulting in semi-quantitative results of high/medium on Truenat and low on GXU.

Finally, 100 mg of stool was defined as the optimum quantity of stool to be used in phase 2 experiments for Truenat. This was based on the fact that in the experiments, volumes of 50–150 mg did not show significant differences in Ct values, and the practicality of visually picking stool would mean that when aiming to pick about 100 mg of stool, in reality, the amount of stool picked would be in the range of 50–150 mg. As 300 mg was problematic, resulting in invalid results on Truenat for subsequent runs, we did not want to increase the quantity.

#### Determining the effect of stool on the Truenat assay

**Fig 4:**
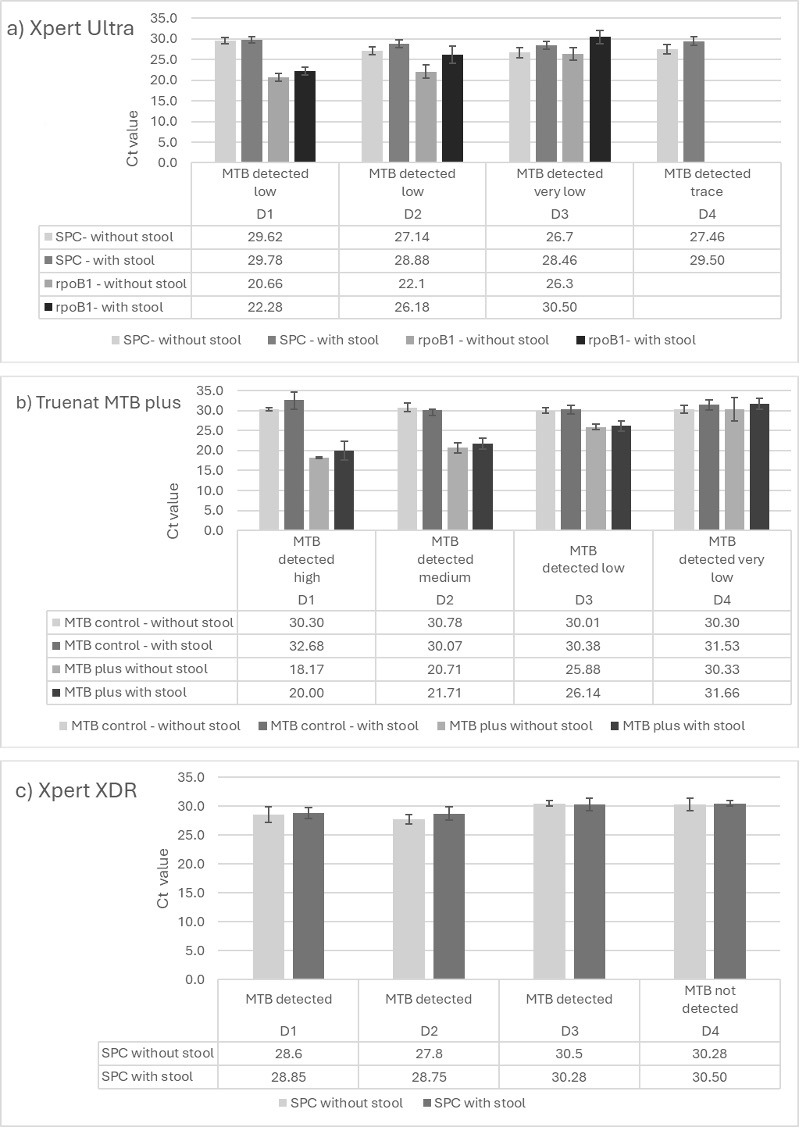
Comparison of Ct values for GXU-rpoB (**a**) and Truenat-MTB Plus (**b**), GXU-SPC (**a**) , Truenat MTB control (**b**), and GXX-SPC (**c**) probe with standard deviation without stool (series B) and with stool added (series C) for the series of MTB dilution suspension. D1 = 5 × 10^6^ cfu; D2 = 5 × 10^5^ cfu; D3 = 5 × 10^4^ cfu; and D4 = 5 × 10^3^ cfu. D, dilution*.*

The Ct values of the Truenat-MTB Plus and GXU-rpoB1 probes obtained from testing the different dilutions of MTB suspension without stool (series B) and with stool (series C) revealed a gradual increase in Ct values with decreasing concentrations of bacteria, as expected for both Truenat and GXU (Fig. 4).

Interestingly, the increase in Ct values upon the addition of stool differed between GXU and Truenat. For GXU-rpoB1, when subtracting the average Ct values between runs with and without stool, the difference was 1.6 for the highest bacterial concentration (D1), and 4.1 for D2 and 4.2 for D3, thus indicating an increase.

This same trend was not observed for Truenat, where the average Ct values of the Truenat-MTB Plus probe with and without stool differed less (1.83 for D1, 1.0 for D2, 0.26 for D3, and 1.33 for D4), see Fig. 4.

#### Determining the effect of stool on the GXX assay

For GXX, the quantity of MTB detected for the different MTB dilutions (D1–D4) did not differ between without stool and with stool. The highest concentration (D1) to the second lowest concentration (D3) resulted in MTB being detected regardless of whether stool was added or not. This was not the case for the lowest MTB dilution (D4), for which three out of five runs without stool and three out of four runs with stool resulted in MTB not being detected. In addition, no significant difference was observed between the Ct values of the SPC-Aphc, indicating that, contrary to GXU, stool might not affect the assay.

### Phase 2—verifying established protocol for stool Truenat and GXX on clinical samples

A leftover stool sample was obtained from 60 presumptive TB patients, see Table 1. In total, 39/60 (65%) had TB confirmed by GXU at the facility, while in 24, MTB was not confirmed by a routine diagnostic test. The majority (40/60) were 15 years and above.

**TABLE 1:** Baseline characteristics of persons whose stool samples were included in phase 2

Characteristics		Number of stools	% of stools
Sex	Male	38	63.3
	Female	22	36.7
Age group	0–5 years	12	20.0
	6–15 years	7	11.7
	>15 years	40	66.7
	Unknown	1	1.7
HIV status	Positive	5	8.3
	Negative	19	31.7
	Unknown	36	60.0
Stool type	Solid	57	95.0
	Liquid	3	5.0
Initial test result at facility	MTB not detected	21	35.0
	MTB detected^*a*^	39	65.0
Total		60	100.0

^*a*^ MTB detected by Xpert MTB/RIF Ultra.

**Fig 5:**
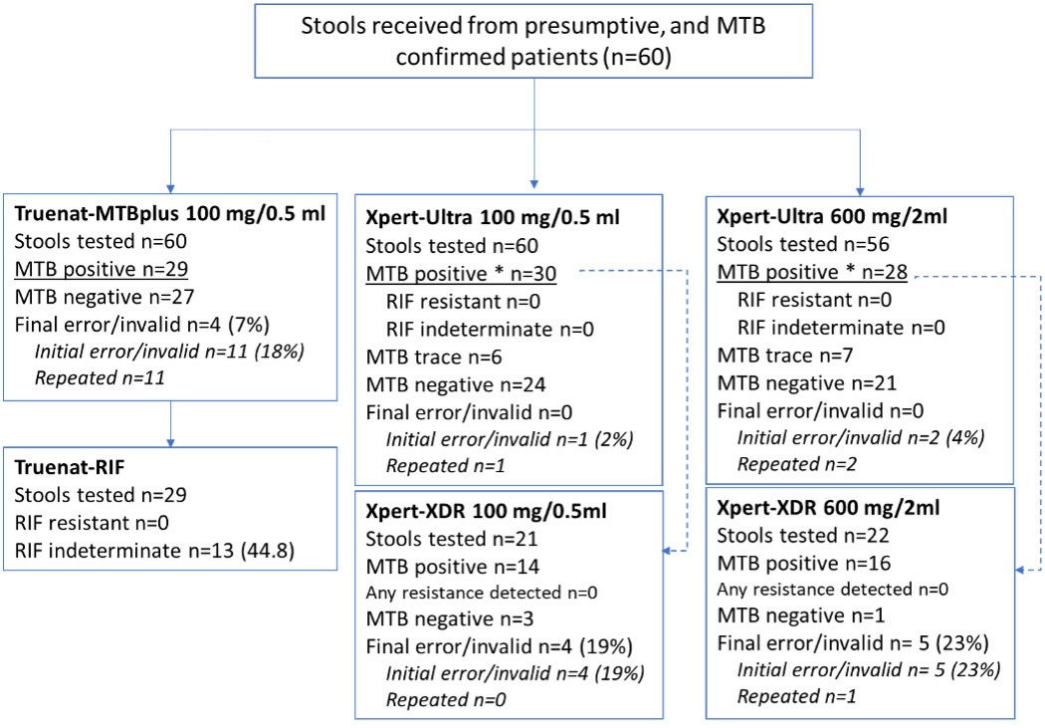
Overview of the results obtained from the comparison of testing the same stool sample using different assays and different quantities of stool. *Final MTB-positive result after repeat testing was conducted and including MTB trace detected. *n,* number.

For all 60 routine stools, a 100 mg stool aliquot was tested using both GXU and Truenat, and for 56 stools, also a 600 mg aliquot was tested using GXU; for the remaining four samples, insufficient stool was left. Of the 35 samples that tested MTB positive on GXU-600 mg, 22 were subsequently tested using GXX, whereas of the 36 MTB-positive samples on GXU-100 mg, 21 were tested using GXX; see Fig. 5. For the remaining samples, not enough material was left.

The proportion of non-determinate test results was higher on Truenat-100 mg than on GXU-100 mg and GXU-600 mg: 11/60 (18%) versus 1/60 (2%) and 2/60 (4%), respectively (*P* < 0.05 for both GXU-100 mg and GXU-600 mg). For Truenat-100 mg, after one repeat test on the same stool sample, 4/11 results remained non-determinate (three invalid [E3] and one error [code 1003]), resulting in a final non-determinate rate of 7%. For GXU, non-determinate results were error code 5006 for GXU-100 mg, and invalid and “No result” for two GXU-600 mg aliquots; after one repeat, all had a valid test result (MTB detected, one low, one trace, and one very low, respectively) (Fig. 5).

**Fig 6:**
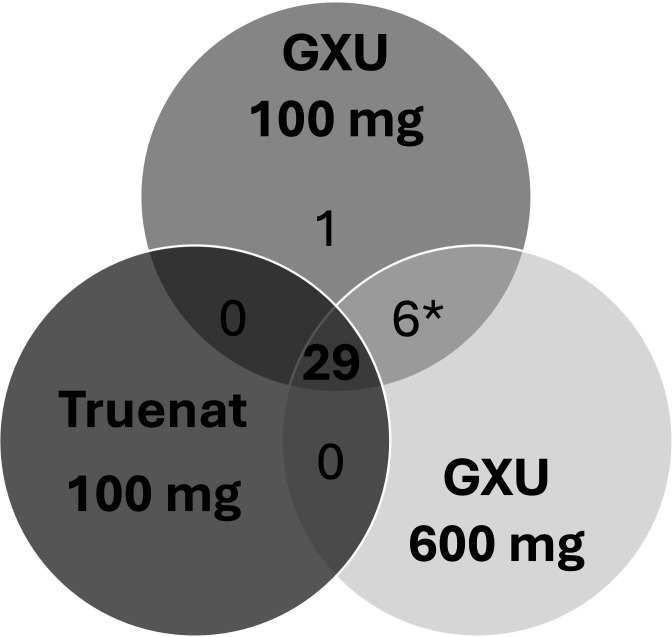
Venn diagram showing the concordance between MTB-positive test results obtained using Xpert MTB/RIF Ultra (GXU) on 100 and 600 mg of stool and Truenat MTB Plus (Truenat) on 100 mg of stool. GXU, Xpert MTB/RIF Ultra; Truenat, Truenat MTB plus; *for one MTB detected on both Xpert Ultra (GXU) 100 and 600 mg, the Truenat results were invalid.

Out of the 60 stools, 35/56 (62.5%) tested MTB positive on GXU-600 mg and 36/60 (60.0%) on GXU-100 mg, while the Truenat-100 mg assay detected MTB in 29/56 (51.8%) samples (Fig. 6). Thus, overall, in 36 stools, MTB was detected by GXU; all of these were also “MTB detected” by GXU in their routine diagnostic test. In addition, there were three samples that were “MTB detected” in the routine test (two MTB detected, low and one MTB detected, very low) but were “MTB not detected” in stool by Truenat and GXU as part of the testing done for this study. All three samples were from participants >15 years of age.

**Fig 7:**
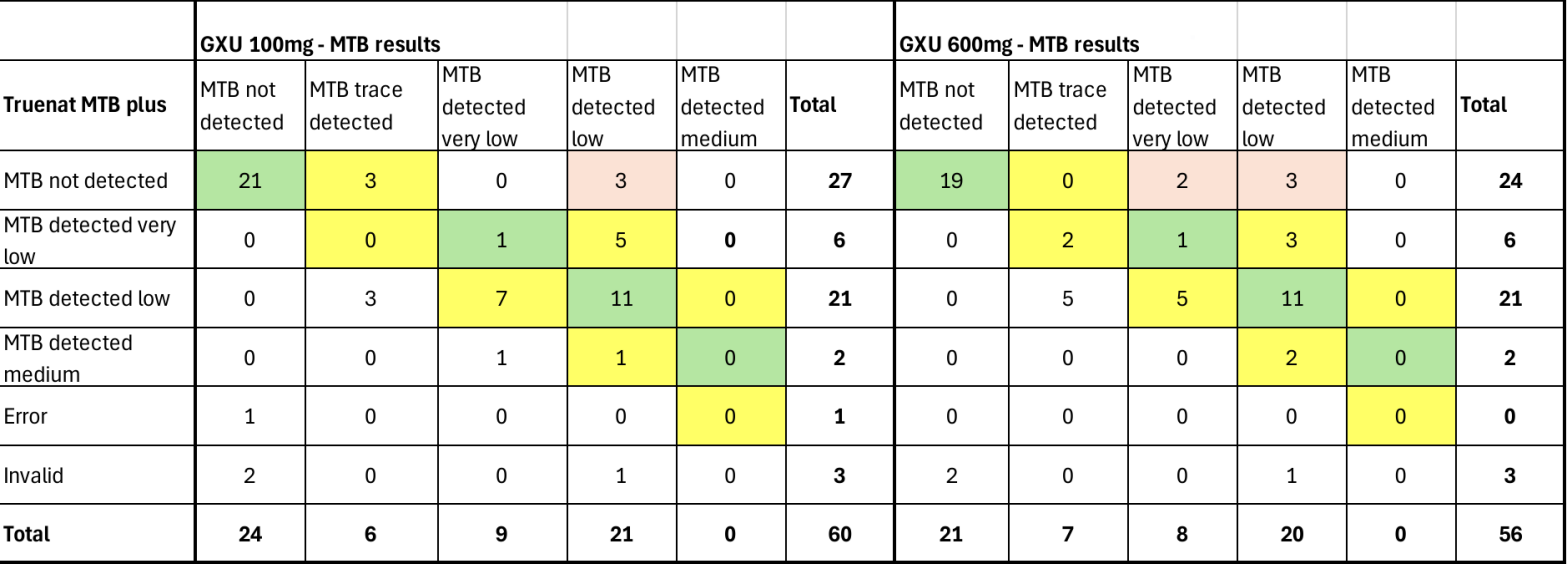
Comparison of semi-quantitative MTB results using Truenat MTB Plus (Truenat) on 100 mg of stool against Xpert MTB/RIF Ultra (GXU) on 100 and 600 mg of stool. GXU, Xpert MTB/RIF Ultra; Truenat, Truenat MTB plus.

The concordance for MTB detection of GXU-100 mg and GXU-600 mg versus Truenat-100 mg was 89.3% (50/56 test results) and 90.6% (48/53 test results), respectively (Fig. 7).

The semi-quantitative results of the six stools, for which the GXU-100 mg result was MTB positive and the Truenat-100 mg result was MTB negative, were MTB detected, trace (*n* = 3) and MTB detected, low (*n* = 3). Five of these were also MTB detected on GXU-600 mg: the three MTB MTB low on GXU-100 mg were also “MTB detected, low” on GXU-600 mg, while two of the three “MTB detected, trace” on GXU-100 mg were “MTB detected, very low” on GXU-600 mg, and one was MTB not detected on GXU-600 mg, which was “MTB detected, trace” on GXU-100 mg. One stool resulted in an invalid result on Truenat-100 mg (even after one repeat test), while its result was “MTB detected, low” on both GXU-100 mg and GXU-600 mg. Concordance between GXU-100 mg and GXU-600 mg was high, with 55/56 (98.2%) results being concordant. Of the 35 specimens that had MTB detected on both GXU-100 mg and GXU-600 mg, 19 (54%) had the same semi-quantitative GXU result, 11 (31%) had a one-level difference in semi-quantitative result (e.g., MTB detected, very low vs MTB detected, low), and 5 (14%) had a two-level difference.

#### Comparison of RIF resistance results between Truenat MTB-RIF Dx and GXU

**Fig 8:**
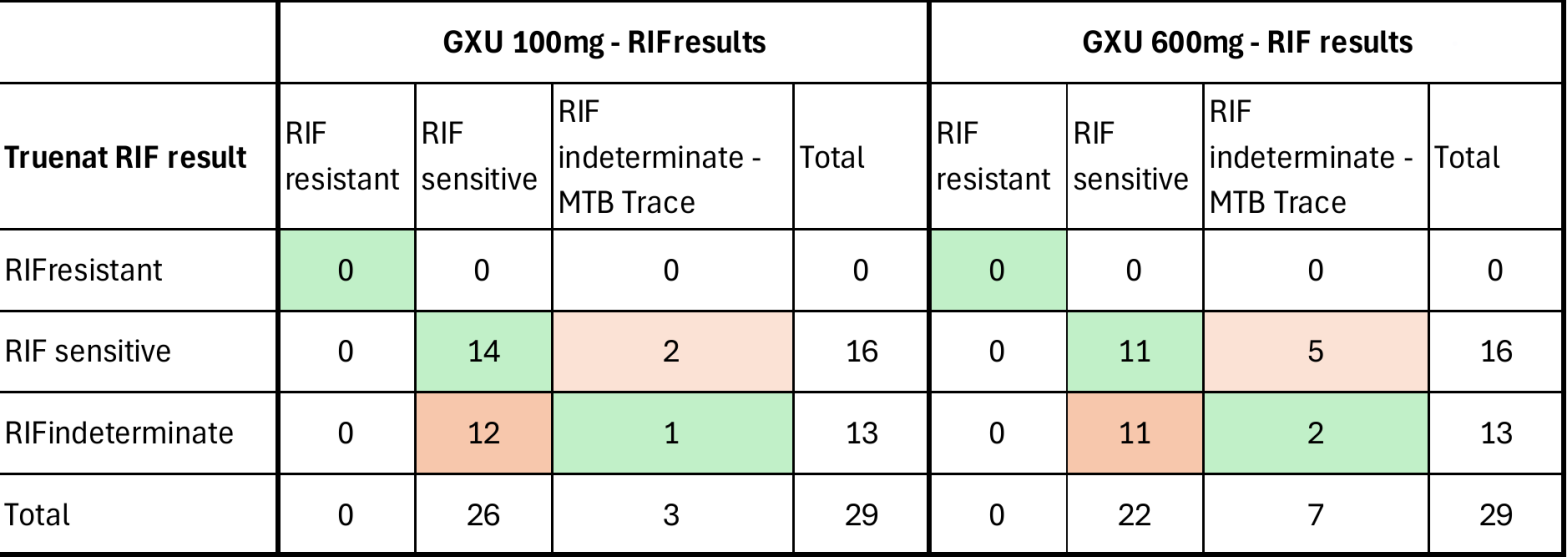
Comparison of results to detect resistance to rifampicin of Truenat MTB-RIF Dx on 100 mg of stool against GXU on 600 mg of stool GXU, Xpert MTB/RIF Ultra; Truenat, Truenat MTB plus.

Figure 8 depicts the comparison between the Truenat MTB-RIF Dx and RIF results from GXU. Truenat MTB-RIF Dx showed a significantly higher RIF indeterminate rate compared to GXU-100 mg (44.8% vs 10.3%; chi-square statistic 8.631; *P* = 0.0033) but not for GXU-600 mg (44.8% vs 24.1%, chi-square statistic 2.7474; *P* = 0.0974). However, all GXU indeterminate results were related to MTB trace detected, which, by default, results in RIF indeterminate due to the probes targeted. Thus, no GXU indeterminate results were observed in samples in which MTB was detected at a semiquantitative load of “MTB detected, very low” and higher.

#### Performance of GXX on stool

GXX-100 mg was conducted for 21/36 samples, and GXX-600 mg was conducted for 22/35 stool specimens, with MTB detected on both GXX 100 and 600 mg, respectively. Using 100 mg of stool, 14/21 results were MTB detected, 3 MTB not detected, and 4 non-determinate (3 invalid and no result). In 600 mg of stool, the results were MTB detected for 16/22 samples, MTB not detected in 1 sample, and 5 results were invalid. Concordance between GXX on 100 and 600 mg was high (15/16 = 93.8% among those with valid results for both tests). There was one sample in which MTB was detected when using 600 mg of stool, but MTB was not detected using 100 mg of stool. In addition, for one specimen, MTB was detected using 600 mg, while a 100 mg aliquot was not tested.

With regard to resistance detection, resistance to isoniazid (INH) was detected in combination with resistance to ethambutol (ETH) for two participants on both GXX-100 mg and GXX-600 mg. In addition, there was one participant with INH and ETH indeterminate results and a different participant with fluoroquinolones indeterminate results.

## DISCUSSION

Our study shows that stool-based testing using the Truenat platform performs well using a slightly modified version of the original SOS stool processing method, adapted to the test composition of the Truenat assay. In addition, we proved the concept that stool can also be tested using the GXX assay using the original SOS stool processing protocol (4). In experimental phase 1, the diagnostic accuracy of Truenat compared to GXU for MTB detection was 100% on 50, 100, and 150 mg of stool. These findings validated the application of the adapted SOS stool processing method to fit the test composition for the Truenat platform.

As expected, only smaller quantities of stool can be used in the Truenat assay compared to GXU, as the volume of the Truenat lysis buffer (2.5 mL) is lower than the 8 mL of the SR buffer used for GXU. Therefore, it was concluded that approximately 100 mg of stool, about the size of a pea, is the optimum quantity for Truenat.

While we previously reported (13) that one may use quantities of 300 mg of stool with GXU for MTB detection, we demonstrate here that it is possible to use as little as 100 mg with both GXU and Truenat. Among routine samples (phase 2), the concordance of test results was 89.3% between GXU-100 mg and Truenat-100 mg, and 90.6% for GXU-600 mg versus Truenat-100 mg. The high concordance of test results (53/54 [98.1%]) between GXU-100 mg and GXU-600 mg confirms that the exact quantity is not so critical. This shows that small quantities of leftover stool are enough to conduct GXX testing as a follow-on test to obtain additional information on the resistance profile of second-line anti-TB drugs. It is known that GXX in sputum has a similar level of detection to the GX assay, which is lower compared to GXU (~112.6 CFU/mL for GX and GXX versus 16 CFU/mL for GXU) (3). This was also observed in our work and needs to be considered. The amount of 100 mg may also provide the opportunity to use rectal or anal swabs instead of stool bowl specimens for quick TB testing in young children and severely ill, hospitalized patients.

In addition, this also confirms that visual picking instead of using a scale to quantify the amount of stool for testing as advised in the SOS stool protocol is justified (13). Further evidence for this is provided by the findings from the experiments in which we determined the optimum stool quantity, which showed no significant difference in the average Ct values of the target probes when stool quantities varied between 50 and 600 mg for GXU, and between 50 and 150 mg for Truenat (series A). Furthermore, the differences in the Ct values between the tests with and without 100 mg of stool (series B and C) were influenced by bacterial load, not by the amount of stool added. The only difference observed in these experiments was that the differences in Ct values of the target probes appeared to increase as bacterial load decreased. Whether this suggests that the inhibition of stool in PCR technology is more pronounced for paucibacillary stool samples needs further investigation.

It is known that with paucibacillary TB samples, different aliquots taken from the same stool sample could result in either MTB-positive or MTB-negative results. This is due to the chance of picking an aliquot that contains sufficient bacilli to reach the test’s limit of detection (16). Therefore, with paucibacillary TB samples, whether an aliquot tests MTB positive is a combination of the amount of bacteria in the specific aliquot and the sensitivity of the specific assay. This is important to consider when interpreting the discordance between MTB detection results for Truenat and GXU on stool. In addition, when comparing the results of Truenat and GXU, a limitation is that it is challenging to directly compare the Ct values. The targets to detect MTB in the GXU assay are rpoB and IS1081-IS6110, and these are provided as separate Ct values for each target probe. For Truenat, however, the targets to detect MTB are nrdZ and IS6110, and the amplification of both targets is reflected in one combined Ct value, called Ct MTB Plus. Furthermore, the limit of detection differs slightly between GXU and Truenat (16 CFU/mL versus 30 CFU/mL, respectively). This needs to be considered when comparing Truenat with GXU.

The initial proportion of non-determinate results for Truenat was 18.3% (11/60), which decreased to 6.7% (4/60) after one repeat test. However, this remains significantly higher than for stool GXU testing, where the non-determinate rate after repeat testing for both 100 and 600 mg was 0%. The most common non-determinate result for Truenat was error code E03, occurring during DNA extraction on the Trueprep AUTO version 2, meaning that no DNA eluate will be available for further testing. Further optimization of the protocol or test kit, such as increasing the volume of lysis buffer, might reduce the non-determinate rate. In addition, increasing the competency of laboratory staff in Truenat stool testing may also help reduce the number of non-determinant test results. Studies from Vietnam, Ethiopia, and the Democratic Republic of the Congo showed that the non-determinate rate for GXU decreased with time when implementing the SOS stool method. This is probably because, even though the test is simple, some experience is required to optimize conducting the test (17, 18) (B. Mujangi, E. Klinkenberg, N. Mintsey, C. Kumakamba, M. Aloni, D. Muteke, et al., unpublished data).

The proportion of RIF indeterminate results obtained with Truenat was unexpectedly high (44.8% [13/29]), while no RIF indeterminate results were observed with GXU, excluding those related to MTB trace detection. This may require further investigation by the manufacturer, as similar results for sputum on Truenat have been reported by Penn-Nicholson and colleagues (8).

Our study had some limitations. First, in phase 1, due to the limited capacity for XDR testing (limited availability of XDR cartridges and just one 10-color module), the XDR assay was excluded from experiment series A. In our opinion, this was justified because the GXX test design is similar to that of the GXU assay, and we therefore anticipated stool to behave similarly on both assays. Of note is also, that we did not compare the GXX resistance results to phenotypic susceptibility testing or another reference; therefore, the performance regarding risk of false positives or false negatives for resistance targets when testing stool specimens on the GXX is unknown. Additionally, during the experiments in phase 1, no valid test results were returned for the liquid stool samples on Truenat, while all three liquid stool samples collected from (presumptive) TB patients in phase 2 returned valid results on Truenat. Possibly, preparing the liquid stool from solid stool disturbed the stool matrix, resulting in smaller particles than those occurring in “natural” liquid stool and poorer sedimentation of debris by gravity.

In phase 2, a limitation was that most routine stool samples tested were from presumptive TB patients aged >15 years, whose samples generally have higher bacillary load than samples from children. In a follow-up study to the current work, we used the adapted SOS stool method for Truenat in a routine setting in Nigeria, including only children, comparing stool on Truenat against stool on GXU (19).

Importantly, the high concordance rate between GXU and Truenat results on stool suggests that childhood TB diagnostic services could be provided in health facilities located in hard-to-reach areas with limited infrastructure, where Truenat is suitable for placement. Our study also shows that more comprehensive drug susceptibility testing using GXX testing can be performed using stool, thus providing access to a follow-on test for children when RIF resistance is detected and the potential for timely diagnosis and appropriate treatment initiation at lower healthcare levels than currently possible. Anecdotal evidence from Ukraine, for example, already indicated that GXX testing can be performed using the same SOS stool-processing method (11). In addition, Khumalo et al. (12) recently reporrted good performance of GXX testing using stool samples in Eswatini. Global scale-up of these alternative testing approaches will be required to help close the gap in childhood TB case finding.

In conclusion, stool can be tested on Truenat to detect MTB using a slightly adapted version of the SOS stool method. The SOS stool method developed for GXU can be used for GXX. This provides opportunities to increase access to rapid molecular testing for children and adults who cannot provide sputum.

## SUPPLEMENTAL MATERIAL

Table S1 jcm.01040-25-s0001.tiff Procedure on how to prepare the series of MTB bacteria suspensions.

ASM does not own the copyrights to Supplemental Material that may be linked to, or accessed through, an article. The authors have granted ASM a non-exclusive, world-wide license to publish the Supplemental Material files. Please contact the corresponding author directly for reuse.
