# Supplementary figures and images for: Evaluation of the Simple One-Step (SOS) stool method for Truenat MTB plus and Xpert MTB/XDR assay
Source: J Clin Microbiol. 2025 Dec 10;64(1):e01040-25. doi: 10.1128/jcm.01040-25 (PMC12802141; doi:10.1128/jcm.01040-25)

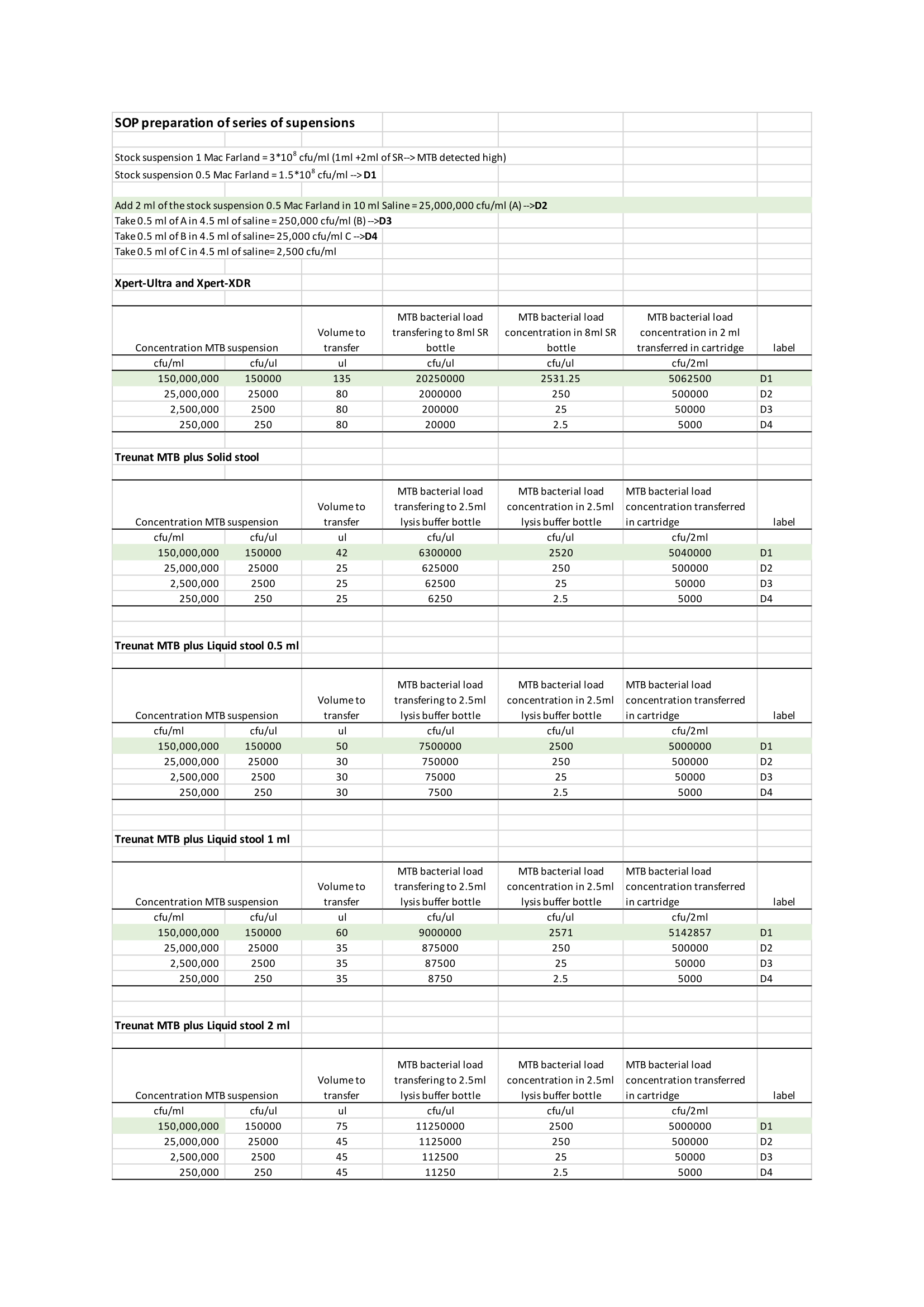

Supplement: Table S1 — Procedure on how to prepare the series of MTB bacteria suspensions. [file jcm.01040-25-s0001.tiff]
